# Relationship of Wine Consumption with Alzheimer’s Disease

**DOI:** 10.3390/nu12010206

**Published:** 2020-01-13

**Authors:** Marcella Reale, Erica Costantini, Srinivas Jagarlapoodi, Haroon Khan, Tarun Belwal, Angelo Cichelli

**Affiliations:** 1 Dept. of Medical, Oral and Biotechnological Sciences, University “G. d’Annunzio” Chieti-Pescara, 65100 Chieti, Italy; Erica.costantini@unich.it (E.C.); Srinivas.Jagarlapoodi@unich.it (S.J.); angelo.cichelli@unich.it (A.C.); 2 Department of Pharmacy, Abdul Wali Khan University, Mardan 23200, Pakistan; haroonkhan@awkum.edu.pk; 3 College of Biosystems Engineering and Food Science, Zhejiang University, Hangzhou 310027, China; tarungbpihed@gmail.com

**Keywords:** alcohol consumption, Alzheimer’s disease, light to moderate wine consumption, neurodegeneration

## Abstract

Background: Alzheimer’s disease (AD), the most threatening neurodegenerative disease, is characterized by the loss of memory and language function, an unbalanced perception of space, and other cognitive and physical manifestations. The pathology of AD is characterized by neuronal loss and the extensive distribution of senile plaques and neurofibrillary tangles (NFTs). The role of environment and the diet in AD is being actively studied, and nutrition is one of the main factors playing a prominent role in the prevention of neurodegenerative diseases. In this context, the relationship between dementia and wine use/abuse has received increased research interest, with varying and often conflicting results. Scope and Approach: With this review, we aimed to critically summarize the main relevant studies to clarify the relationship between wine drinking and AD, as well as how frequency and/or amount of drinking may influence the effects. Key Findings and Conclusions: Overall, based on the interpretation of various studies, no definitive results highlight if light to moderate alcohol drinking is detrimental to cognition and dementia, or if alcohol intake could reduce risk of developing AD.

## 1. Introduction

Throughout history, wine has often been used as a tool to manage health, and has been prescribed as medicine to treat symptoms or to promote health by preventing the most common ailments. In Mesopotamia, wine was used in various treatments and therapies, and was mixed with honey as a common therapy for cough treatment. The Egyptians used wine as a solvent, a remedy for weakness and injuries, or as an appetizer. The ancient Greek physician Hippocrates considered wine to be a key nutrient of a healthy diet and prescribed its use as a sedative and antiseptic [1]. Although many beneficial and negative effects of alcohol on health have been highlighted, controversies remain about the properties wine components and their cellular and molecular effects. The effect of wine on health is dose-dependent and the border between the quantity causing problems and the quantity that could be beneficial to health is low.

Drinking alcohol is a behavior that is increasing in popularity, especially among young people. Many people consume alcohol conscientiously and without health consequences, but the number of those who harmfully drink alcohol is growing. Many different kinds of alcoholic drinks exist with different alcohol contents. Although alcohol is legal and widely available, drinks with higher concentrations of alcohol risk causing health problems more quickly and in smaller doses.

**Figure 1:**
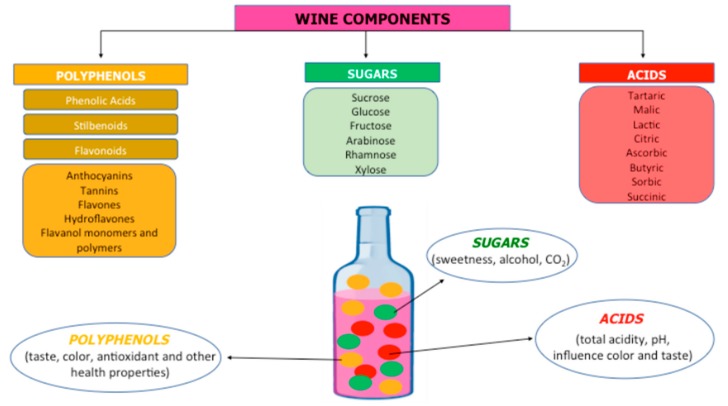
The several components of the wine.

Wine contains the products of the must or juice of the fruit of the species *Vitis vinifera* fermented by yeast. After fermentation, wine contains a complex mixture of different compounds affecting its quality. In alcoholic wine, phenolic compounds, polysaccharides, acids, volatile compounds, and water are present in varying concentrations. The principal polyphenols are flavanols, flavonols, anthocyanins, and resveratrol; flavonoids include catechin, epicatechin, proanthocyanidins, flavones, and anthocyanins [2]; and up to 60% of total phenolic compounds are represented by catechin and epicatechin, producing an antioxidant activity related to the inhibition of nuclear factor kappa-B (NF-κB) and inflammatory cytokines. Myricetin, kaempferol, rutin, and quercetin are flavonols, and quercetin provides ability to induce the activity of antioxidant enzymes such as heme oxygenase, glutathione S-transferase, and thioredoxin reductase to inhibit NF-κB translocation to the nucleus and to reduce the expression of toll-like receptors (TLR2 and TLR4), thus creating its anti-inflammatory activity [3]. Delphinidin-3-glucoside, cyanidin-3-glucoside, and malvidin-3-glucoside are the most commonly found anthocyanins in wines, with antioxidant and anti-inflammatory activities, along with resveratrol, which is able to induce or repress inflammatory cytokines, such as tumor necrosis factor-(TNF)-α, interleukin- (IL)-1β, and IL-6 modulating the inflammatory response, and inhibiting NF-κB and inflammatory enzymes, such as the inducible isoforms of nitric oxide synthase (iNOS) and cyclooxygenase-1 (COX-1) [4]. Tannin is present in the skins and seeds of grapes, which is another subgroup of phenols responsible for the quality of wine, contributing to color, bitterness, astringency, and structure of wine. Wine polyphenols, by activation of antioxidant and anti-inflammatory mechanisms, possess beneficial and therapeutic properties for prevention of neurodegeneration, cancer, metabolic disorders, and aging, as demonstrated by preclinical and in vitro studies. Polyphenols also play an important role in the treatment of pathogen infection, hypertension, and cardiovascular diseases [5,6,7,8]. The most abundant sugars present in grapes are glucose and fructose, whereas sucrose is only present in trace amounts. Ethanol and carbon dioxide are produced through the breakdown of sugars by yeast in the process of fermentation. The volatility of aromatic compounds is related to sugar concentrations. Sweetness, which is influenced by ethanol, acids, and tannins, is detected at levels higher than 1% (*w*/*v*) of overall sugars. Organic acids, such as tartaric, malic, and to a lesser extent, citric acids, are the most abundant solids present in wine and are responsible for the taste, wine stability, color, and pH. Sun et al. identified 2-O-feruloyl tartaric acid as potential phosphodiesterase 4D inhibitor (PDE4D). PDE4D alters the function of calcium channels in the central nervous system and is considered one of the causes of Alzheimer’s disease (AD) [9]. Low concentrations of proteins in wines are responsible for the clarity and stability of wines in association with factors of non-protein origin, such as polyphenols, pH, and polysaccharides. Nitrogenous compounds include ammonium cations and amino acids, peptides, and proteins are nutrients for yeast and lactic acid bacteria. The most important mineral compounds in the wine are potassium, sulfate, phosphates, sodium, iron, and chloride [10,11,12]. The composition of wine is summarized in Figure 1, and, with any natural biological material, the components may vary significantly.

Consumption of wine is one component of the Mediterranean diet, and is increasingly associated with the promotion of human health and the prevention of diseases mainly associated with mental and heart health. However, possible health benefits may only exist with moderate drinking, i.e., “Up to one drink per day for women and up to two drinks per day for men and only for adults of legal drinking age” as reported by Dietary Guidelines of the United States (2015) [13]. The National Institute on Alcohol Abuse and Alcoholism (NIAAA) defines moderate drinking as up to four alcoholic drinks for men and three for women in any single day and a maximum of 14 drinks for men and seven drinks for women per week. Moderate wine drinking is linked to higher blood levels of omega-3 fatty acids that protect against heart disease, metabolize glucose, and decrease cardiometabolic risk; increased levels of heme-oxygenase and prevention of blood clotting may protect the brain from stroke damage [14,15,16,17]. The risk of developing dementia and depression was suggested to be reduced by moderate wine drinking [18,19].

In the elderly, wine is a commonly consumed alcoholic beverage, especially during meals, whereas other alcoholic drinks are only drunk occasionally. Research has indicated that the positive effects of red wine on health are based on the presence of antioxidants that attack free radicals via different mechanisms. The antioxidant potential of red wine has been highlighted by the results of the French paradox, which describes how, in the French population, despite a relatively high dietary intake of saturated fats, the incidence of cardiovascular disease is relatively low, probably linked to the consumption of red wine [17]. The French paradox may have its basis in an environment containing various important molecules, and the advantages can be mainly linked to the joint, cumulative, or synergistic effects of alcohol with other components in wine.

Researchers are working to discover the relationship between the bioavailability of >200 phenolic compounds present in wine and their molecular and nutritional properties, environmental, social, or family factors that influence wine consumption, and the effects of alcohol on health. Although the benefits of polyphenols in fruit and vegetables are increasingly accepted, how wine, and in particular red wine with its abundant content of phenolic acids and polyphenols, provides further health benefits has not yet been fully clarified [20,21].

Many of the disorders due to alcohol intake are related to large amounts of consumed alcohol. About 10% of the population drinks 75% of the alcohol consumed in the United States [22]. Moderate wine drinking may have some health benefits; excessive alcohol consumption increases the risk of several cancers, heart disease, other chronic diseases, and mental health problems. Chronic alcohol consumption results in persistent changes in the brain, driving the reduction of behavioral control and difficulty avoiding negative consequences. Alcohol has been examined as a risk factor of dementia, and a relationship between alcohol consumption and dementia has been evidenced. Although chronic alcohol abuse results in significant activation of neurodegenerative processes [23] and the prevalence of alcohol-related dementia represents about 10% of all cases of dementia [24], whether light-to-moderate drinking has any health benefits remains to be determined [25]. The relationship between the amount of alcohol intake and cognitive outcomes is complicated by differing definitions of high levels of alcohol consumption, by the duration of abuse, the age at which alcohol consumption begins, sex, and alternation of binge and withdrawal periods.

Since a link between alcohol misuse and AD has been described, and alcohol dependence is a significant and independent predictor of AD [26], this review was designed to examine the association between wine intake and AD.

## 2. Neurodegeneration and Alzheimer’s Disease

Neurodegenerative diseases are age-dependent disorders with increasing prevalence, partly due to increased life length [27]. Neurodegenerative diseases are a group of diseases in which the loss of neuronal cells in certain areas of the brain is irreversible and progressive, related to loss of movement cognitive and behavioral function. The main neurodegenerative diseases include Alzheimer’s disease, Parkinson’s disease, amyotrophic lateral sclerosis, and Huntington’s disease, joined by some pathogenic and clinical characteristics. Dementia is responsible for the main burden of neurodegenerative diseases, and Alzheimer’s represents approximately 60–70% of dementia cases. According to the World Alzheimer’s Report 2016, 46.8 million people in 2015 were affected by dementia, and an increase of up to 131.5 million is expected by 2050. Based on the neuropsychiatric assessment of clinical features and memory deficits, diagnosing only probable AD is possible; certain diagnosis is only possible post-mortem with the examination of cerebral degeneration [28].

Several factors, including genetic and environmental factors, affect neurodegenerative diseases onset. Risk of AD development is associated with factors such as apolipoprotein-E (APO-E), diabetes mellitus, aging, smoking habits, and lower socio-economic status. Decreased risk of AD onset appears to be related with physically and cognitively stimulating activities, adherence to the Mediterranean diet, light-to-moderate alcohol consumption, and high level of education [29]. In the brain, the build-up of toxic proteins and a loss of mitochondrial function are the events that are precursors to progressive neuronal damage. Oxidative stress, linked to direct neuroinflammation, metal accumulation, and mitochondrial dysfunction, plays a crucial role in neurodegeneration [30,31]. The cascade of pathological events linked to neurodegeneration is characterized by a high production of free radicals that act on nucleic acids, proteins, and fats, as well as on glycosylation of DNA and proteins, inducing apoptosis and necrosis. Several proteins that undergo structural and functional changes responsible for degradation and misfolding, such as β-amyloid (Aβ) in Alzheimer’s disease and α-synuclein in Parkinson’s disease [32], may be present in small quantities in elderly subjects. Oxidative stress elicits lipid peroxidation, and derived products react with protein to form intracellular precipitates; oxidative stress also induces activation of glial cells that release pro-inflammatory cytokines strengthening the neurodegeneration.

AD, a progressive and neurodegenerative disease, affects more than 5% of the population over the age of 65 years. The neuropathology of AD is characterized by the presence intraneuronal deposits of neurofibrillary tangles, senile plaques around reactive microglia, deposits of amyloid β (Aβ), and progressive loss of neurons and brain functions [33]. Neuronal death may be responsible for memory failure, personality changes, and other manifestations. Brain inflammation is another hallmark of AD [34], being present from pre-clinical to terminal stages of the disease, as indicated by activated microglia and reactive astrocytes that edge plaques and secrete cytokine, thus increasing the neuro-inflammatory response [35]. The central role of inflammation in AD has been highlighted by many studies, confirming that AD pathogenesis is not restricted to the neuronal compartment, but also involves the central and peripheral immune systems. High levels of pro-inflammatory cytokines, such as IL-1β, IL-6, TNF-α, transforming growth factor (TGF)-β, and IL-18, have been found in the brain, cerebrospinal fluid, and peripheral blood, suggesting that AD may be associated with a more widespread inflammatory state [36,37,38].

Since the late 1990s, accumulation of Aβ and the deposition of neurofibrillary tangles have been considered the main causes of AD [39,40]. More recently, alternative explanations of the pathogenesis of AD have emerged, such as oxidative damage increasing production of reactive oxygen species (ROS) [41], loss of mitochondrial function [42], altered metal homeostasis, and reduced antioxidant defense [43], which could influence the production and accumulation of Aβ and hyperphosphorylated Tau protein, driving a vicious cycle that could worsen mitochondrial dysfunction and ROS production. Acetylcholinesterase (AChE) expression is substantially altered and its activity increases within and around the Aβ plaques but decreases in most brain regions of AD patients. Many studies are being performed to examine the relationship between obesity and neurodegenerative diseases and one of the major risk factors for dementia is metabolic syndrome and abdominal obesity, correlated with deregulation of adipokines [44,45].

AD is evidently a complex multifactorial syndrome and a general agreement exists that factors like smoking, physical exercise, lifestyle, diet, and education may play a central role in the AD.

## 3. Intake of Alcoholic Beverages: Risk or Protection of Alzheimer’s Disease?

Albeit through different mechanisms, both no consumption or excessive consumption of alcohol are both associated with an increased risk of dementia [46]. Deng et al. identified the relationship between drinking and risk of dementia, proving that elevated risk of cognitive impairment and high consumption of alcohol are related, and reduced risk of dementia is related to light-to-moderate alcohol drinking [47].

Xu et al. reported a J-shaped relationship between alcohol intake and cognitive decline in patients with mild cognitive impairment, and showed that high consumption of alcoholic beverages as well as complete abstention increase the risk of dementia, and that light–moderate alcohol drinking may be associated with a decreased risk of dementia [48]. Many studies clarified if alcohol may be a risk or protective factor of developing AD, but the evidence is far from conclusive and the associations between abstinence, moderate, or heavy drinking and risk of dementia remain unclear.

In general, the literature examining the impact of alcohol on AD shows several limitations such as the validity of measurement of alcohol consumption, period of time (day, month), consumption (continuous or variable), and the cognitive assessments of drinkers. Environmental, socio-economic, and lifestyles factors, together with unknown causes of AD, can contribute to the uncertainty of the role of wine intake in AD. Cumulative evidence reveals that intermittent ethanol intake during adolescence enhances the weakness of the brain to both ethanol-induced neural cell death and cognitive impairments. Studies on human alcoholic brain found a correlation between the rate and amount of lifetime alcohol consumption and whole brain damage and reduction in the number of neurons. Most studies did not distinguish amongst wine, beer, or spirits, and studies that did distinguish reported no difference among the effects of these different types of alcohol. Thus, only further and in-depth studies will clarify the intricate relationship between alcohol consumption and the onset of AD and clarify the risks or benefits involved in alcohol consumption, and if and how diet and ingestion of wine in particular may help slow the cognitive decline and protect against AD regardless of other risk factors.

### 3.1. Moderate Alcohol Consumption

Evidence exists that moderate consumption of wine positively affects organs and systems. Using rodent models, cardiac myocytes, and endothelial cells, the results of several studies indicated that moderate alcohol intake can support anti-inflammatory processes involving adenosine receptors, protein kinase C (PKC), and nitric oxide synthase, which could drive cardioprotection. Collis et al. reported that the modifications to alcohol-related anti-inflammatory heat shock protein and protein kinase in the brain have similarities with those observed in the heart [49], highlighting that dementia and cardiovascular disease share several common risk factors. These observations have encouraged studies on the association of alcohol intake with dementia. An inverse correlation between cardiovascular risk and moderate alcohol intake has been highlighted, which is nullified when consumption is high. Several systemic reviews were conducted to answer the question “Is alcohol consumption a protective factor against AD?” Taken together, the literature does not provide adequate evidence for a conclusive answer. A study reported that light-to-moderate/regular drinking have a protective effect against AD [47], whereas other studies reported a protective effect of moderate-to-high levels of drinking, but many variables, such as age and sex, ethnicity, measures of alcohol, clinical evaluation, and use of standardized cognitive assessments, do not allow drawing adequate conclusions [50,51,52,53,54,55]. Zuccalà et al. showed that cognitive dysfunction is proportionally associated with high or moderate alcohol consumption and sex [56]. A longitudinal study examined the association between alcohol consumption and dementia and showed that middle-aged non-drinkers have a greater risk of developing dementia than those who drink moderate amounts, with more pronounced effects particularly evident in people who drink wine. Cohort studies have indicated that light or moderate alcohol consumption may reduce or not significantly change the risk for AD due to the presence of resveratrol in red wine [57,58]. No evidence exists that consumption of alcohol between 1 and 14 units/week (1 unit = 10 mL or 8 g pure alcohol) increases the risk of dementia, whereas >14 units/week linearly increases the risk of dementia with age. Thus, risk of developing dementia progressively increases as alcohol drinking increases. Even though men and women qualitatively and quantitatively consume different amounts of alcohol, no sex differences in alcohol effects on cognition were observed [59]. The Rotterdam study reported that in individuals aged from 55 years and older, drinking a light to moderate amount of alcohol (1–3 drinks per day) is correlated with a lower risk of dementia and that this outcome is not especially confined to any beverage type [60]. Others European investigations reported that a modest alcohol intake is correlated with a reduced risk of dementia [61,62].

In several studies, drinkers have been compared with non-drinkers, and results showed that those who drink a light-to-moderate amount of wine exhibited lower lifetime risk for AD than those who abstained, and that both non-heavy drinkers and abstainers exhibited a decreased risk of AD [63]. The effect of moderate alcohol consumption for cognition was found in both men and women, although the amount and timing of drinking is different between men and women. Overall, in younger subjects, cognition does not appear to be compromised by light to moderate drinking whereas in older subjects, mild to moderate drinking seems to reduce the risk of dementia and cognitive decline.

The study of a cohort of individuals aged 65 and over showed that light or moderate alcohol drinking is associated with a lower risk of dementia and AD, whereas beer and liquor drinking is not associated with risk of dementia. The analyses were stratified according to the presence of Apolipoprotein E (APOE)-4 and showed that the association between the light to moderate wine intake and A lower risk of AD was limited to subjects without the APOE-4 allele. Thus, reduced cognitive risk effect of moderate drinking was annulled by the presence of the APOE-4 allele [58]. A study on binge Finnish drinkers without the APOE-4 allele reported an insignificantly reduced risk of dementia, whereas infrequent binge drinkers with the APOE-4 allele displayed significant increase in the risk of dementia [64]. In a study conducted to analyze the effect of alcohol and APOE-4 on the age of onset of AD, the absence of the APOE-4 allele in drinkers was found to be associated with an earlier onset of AD [65]. Women who drink moderately had a significantly reduced risk of cognitive decline independent of APOE-4 [66]. The question of whether the APOE-4 allele influences the protective effect of moderate ethanol intake on cognitive risk has not yet been resolved [58]. A glymphatic system [67] formed by astroglial cells was discovered in the central nervous system, which may eliminate soluble proteins and metabolites and plays a key role in the clearance of proteins that are potentially neurotoxic, such as Aβ18 and Tau23. The effect of acute and chronic exposure to ethanol on glymphatic function was investigated. A reduction in glymphatic function resulting from acute and chronic binge alcohol intake and, surprisingly, in mice treated with a low acute dose or a low chronic dose of alcohol, an increased glymphatic activity was observed [68]. Thus, the increase in glymphatic function combined with the reduction of glial fibrillary acidic protein expression could play an important role in the reduction of risk of dementia in individuals who habitually intake low amounts of alcohol.

All studies confirm that when light-to-moderate wine drinking is associated with a healthy lifestyle and habits, such as the adoption of the Mediterranean diet and physical activity, the positive effects are even more pronounced.

Animal models have shown that the synapse damage induced by amyloid-β and α-synuclein may be counteracted by low concentrations of alcohol [69], and polyphenols, due to their antioxidant properties, might provide neuroprotection.

### 3.2. High Wine Consumption

Chronic intake of alcohol is linked, other than to cardiac and liver problems, to cognitive impairments and brain damage. The characteristics of dementia due to excessive alcohol drinking have received increased interest, and both neuropathological and imaging studies have suggested that excessive and prolonged use of alcohol may be responsible for structural and functional brain damage [70,71,72]. Chronic or excessive alcohol consumption may cause damage to the temporal lobe similar to that observed in AD [73]. Loss of white matter in the prefrontal cortex, corpus callosum, and cerebellum, and neuronal loss in the hypothalamus and cerebellum was observed [74].

Cholinergic dysfunction and neuroinflammation are characteristic hallmarks of dementia, as confirmed by the ability of acetylcholine (ACh) receptor agonists or AChE inhibitors to improve cognitive functions and decrease the levels of inflammatory cytokines in AD patients. Ethanol intake reduces expression of Choline acetyltransferase (ChAT), induces loss of ChAT+ neurons, upregulates neuroimmune signaling, such as proinflammatory cytokines and their receptors, and increases of NF-κB p65 (pNF-κB p65) phosphorylation, in association with increased neuroimmune signaling [75]. Chronic alcohol use has been linked with degeneration of cholinergic neurons, as confirmed by the positive effect of pharmacological manipulation of the neuronal cholinergic pathway [76]. Alcohol may affect cognition by modulating the synthesis and release of acetylcholine in hippocampus, and may induce muscarinic and benzodiazepine receptor loss contributing to the cognitive deficits in AD [77,78]. Thus, one mechanism through which alcohol intake could be linked to AD is alcohol’s effect on cholinergic system.

In AD, most risk loci are located in or near genes that are predominantly expressed in microglia, confirming the hypothesis that microglia play a decisive role in AD progression. Microglial activation was detected in the brains of human alcoholics, but the debate whether microglial activation is the cause or consequence of alcohol-induced neurodegeneration remains open [79]. Toll-like receptors (TLRs), high-mobility group box 1 (HMGB1), microRNAs, and pro-inflammatory cytokines and their receptors are involved in signaling between microglia, innate immune cells of the brain, and neurons in response to alcohol. Ethanol, at high concentrations, is capable of activating TLR4 signaling in astrocytes and microglia [80,81], and triggering the production of inflammatory mediators induces neuronal death. Ethanol can activate microglial cells, altering their morphology, phagocytic response, and production of inflammatory cytokines, such as TNF-α and IL-1β, or inflammatory mediators such as nitric oxide (NO), driving neurodegeneration. A study reported that ethanol, at relevant concentrations, induces microglia activation and secretion of cytokines and inflammatory mediators [81]. Chronic ethanol treatment increases the production of cytokines and inflammatory mediators and induces neural cell death [82]. Several studies have demonstrated that a conditioned medium of ethanol-treated microglia induces apoptosis in cultured neurons, in agreement with observations that high levels of inflammatory mediators produced by activated microglia are deleterious for neurons [83,84,85]. Ethanol induces upregulation of COX-2 and iNOS expression NO production in glial cells, [80,86], and triggers signaling pathways that prime the production and expression of cytokines and inflammatory mediators and cell death in the brain [82].

Taken together, the results of numerous studies indicate that neuroimmune signaling plays a key role in the development of alcohol-related brain disorders. Chronic ethanol sensitizes both systemic and brain responses to the neuroimmune-gene, resulting in the hypothalamic-pituitary-adrenal (HPA)-mediated enhancement of peripheral cytokines, which further exacerbates the neuroimmune response, which increases neurodegeneration [87,88,89,90]. In addition to neuroimmune signaling, glutamate excitotoxicity also is linked to alcoholic neurodegeneration. Neuronal degeneration observed in the adult brain after chronic alcohol exposure gave rise to the neurotoxicity hypothesis, according to which chronic intake of alcohol can cause glutamate excitotoxicity and oxidative stress with neuronal loss [91]. If excitotoxicity is fundamental for neuro-destruction in adult models of chronic alcoholism is only speculative, further studies are needed [92].

The effect of alcohol is also related to age; subjects who begin to consume alcohol before the age of 20 years demonstrated more serious deficits on multiple memory tasks than those who started drinking after the age of 20 [93]. Heavy drinking during adolescence is associated with damage to the prefrontal cortex and hippocampal area, and with neurocognitive dysfunctions as well as in visuospatial, verbal, and attention functions [94,95]. Pascual et al. showed that, during adolescence, intermittent ethanol intake induces inflammation and cell death in the neocortex, hippocampus, and cerebellum, and cognitive impairment, supporting the role of inflammation in ethanol-induced brain damage [96]. Overall, alcohol may support the generation and sustenance of AD pathology via neuroinflammation.

Studies on animals demonstrated that prolonged intake of alcohol induces alteration in different areas of brain such as the hippocampus, hypothalamus, and cerebellum, with impairment of cholinergic neurotransmission, which plays a key role in attention, learning, and memory [97]. The effects of chronic alcohol intake on both basal forebrain cholinergic nuclei and on the brainstem cholinergic nuclei have been studied. The results showed a reduction in the expression of choline acetyltransferase (ChAT), which is involved in acetylcholine biosynthesis [98,99]. Several studies showed that reduction of ChAT is correlated with memory and cognitive impairments in patients with AD and other neurodegenerative diseases [100,101]. A critical role of the TLR4 response was confirmed by observation of microglia activation and neuroinflammatory damage induced by ethanol, and by microglia activation (CD11b^+^ cells) in cerebral cortex of TLR4^+/+^mice, but not in TLR4^-^deficient mice after acute ethanol administration. Fernandez-Lizarbe et al. [81] demonstrated that a conditioned medium of ethanol-treated microglia induces apoptosis in cultured neurons. Growing evidence suggests that, in rats, alcohol-intake-related alterations in brain circuitry are linked to neuroimmune signaling via TLRs [102]. Qin et al. showed that both 10 daily doses and an acute dose of ethanol induce a persistent increase in proinflammatory cytokines and microglial activation in the mouse brain [103].

## 4. Effects of Components of Wine on AD Molecular Targets

Although most studies reported an insignificant association between risk of AD and wine intake, this does not necessarily mean that alcohol has no effect. The mechanisms through which wine intake may influence the risk of developing AD are not completely understood.

Whether the effect of wine on health is attributed to ethanol, micro-components of wine, or their synergistic effect is not yet known. Distinguishing the action of one from that of the other micro-components of wine is difficult. Even the use of alcohol-free wine confounds this issue because the bioavailability of the wine compounds can change in the absence of ethanol.

**Figure 2:**
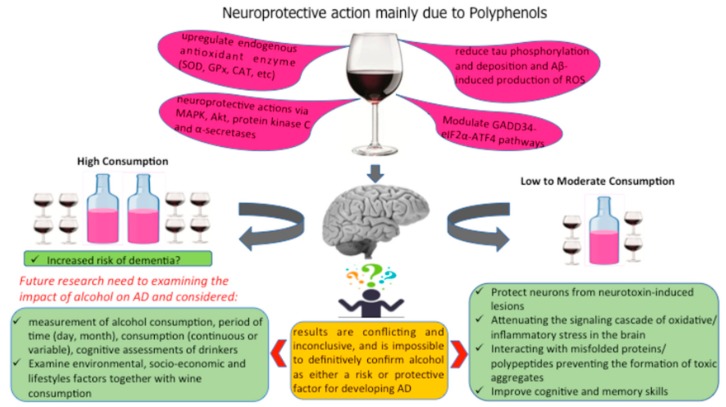
Different components of wine that may protect the brain.

Alcohol’s possible beneficial effects are attributed principally to polyphenols. Useful outcomes of polyphenols on AD have been principally reported in investigations with animal models for AD, considering the effects of fortified diets in specific polyphenols such as resveratrol, epigallocatechin-3-gallate, and quercetin [104,105,106] or in a mixture of polyphenols viz. grape seed extracts and red wine [107,108,109,110]. Dietary polyphenols including those present in grape products are considered promising neuromodulatory agents to fight AD because they have the ability to cross the blood–brain barrier, to protect neurons from neurotoxin-induced lesions, to weaken the signaling cascade of oxidative stress and inflammatory response in the brain, to interact with misfolded proteins and polypeptides preventing the formation of toxic aggregates, and to improve cognitive and memory skills (Figure 2) [111].

Several studies showed that the suggested health benefits from red wine consumption are due to resveratrol, whose amount in red wine can vary widely. A neuroprotective effect of resveratrol was suggested on neuron cell death, induced by ethanol and other oxidative agents [112], as well as a powerful neuroprotective activity in focal cerebral ischemia [113] and antioxidant and ion channel regulation (Ca2þ channels). Antonio et al. reported that resveratrol protects against ethanol-induced neurotoxicity [114]. Resveratrol has several effects on AD pathogenesis, targeting many molecular mechanisms of the amyloid cascade such as inhibition of Aβ fibrils formation [115,116], reduction of Aβ production through sirtuin-dependent activation of a disintegrin, and metalloproteinase domain-containing protein 10 [117], and autophagic and lysosomal Aβ degradation [118].

Resveratrol may interrupt the amyloid cascade acting as antioxidant and anti-inflammatory agent, reducing tau phosphorylation and deposition as well as Aβ-induced production of reactive oxygen species (ROS) [119]. Other than the free-radical scavenging abilities [120], resveratrol shows the ability to upregulate endogenous antioxidant enzymes, such as superoxide dismutase (SOD), glutathione peroxidase (GPx), catalase (CAT), and heme oxygenase, and to downregulate enzymes involved in the production of ROS, such as xanthine oxidase [121]. Resveratrol is able to counteract the production of mitochondrial ROS and effectively eliminate hydroxyl, superoxide, and metal-induced radicals, with resulting strengthening of mitochondrial activity and biogenesis by the activation of the Sirtuin1- Peroxisome proliferator-activated receptor gamma, coactivator 1 α (SIRT1-PGC-1α) pathway, thus intensifying mitochondrial bioenergetic productivity [122,123,124]. Sirtuin-1 (SIRT1), activated by resveratrol, induces direct deacetylation of tau to acetylate, helping its proteasomal degradation [125]. Phospho-tau toxicity (induced by cyclin-dependent kinase 5-p25-dependent tau phosphorylation) may decrease; thus, the deacetylation of (PGC-1α, and p53 is promoted.

Resveratrol showed the ability to reduce the inflammatory status [126,127] in in vitro and in vivo settings of neuroinflammation by inhibiting both IL-8 and granulocyte-macrophage colony-stimulating factor release, cytokine-stimulated iNOS expression, and the development of cytokine-producing CD4^+^ and CD8^+^ T cells by peripheral blood mononuclear cells [128,129].

Resveratrol interacts with several proteins and pathways involved in the pathogenesis of obesity, such as mitochondrial ATP synthase and complex III, fatty acid synthase, protein kinase C, p53, a protein kinase activated by mitogen 1, TNF-α, and NF-κB. Milne et al. reported that various resveratrol analogs are able to reduce insulin resistance by improving energy homeostasis [130]. In diabetic mice, the activation of AMP kinase by resveratrol protects against atherosclerosis and liver damage, in agreement with the observations of De La Lastra et al. [131]. Aruoma et al. [132] reported that oligonol, a low-molecular-weight proanthocyanidin dietary biofactor, exhibited neuroprotective effects by modulating oxidative stress and additional factors [133].

Using the 3xTg-AD mouse model of AD, characterized by learning and memory deficits, the administration of a low dose of a selected pool of polyphenolic compounds of wine slowed disease progression without undesired side effects on healthy controls, as suggested by the results obtained with non-Tg mice. Starting from the observation that polyphenolic-enriched nutrition may have the potential to benefit AD patients by modulating multiple disease-modifying mechanisms, the development of polyphenolic compounds may provide an alternative strategy for treatment and/or prevention of AD [134].

Quercetin is found in wine, affecting its color and taste. In addition to this function, quercetin is a molecule representative of flavonols, which have many beneficial effects on human health such as reduction of the risk of atherosclerosis by reducing low-density lipoprotein, and reduction of IL-1β, C-reactive protein, and monocyte chemotactic protein-1 levels. Quercetin has been identified as one of the potent antioxidants, and AD can be benefited by the effective removal of ROS. Quercetin induces a decrease in oxidative stress by increasing glutathione (GSH) levels in astrocytes and neurons, which is probably responsible for the reduction of Aβ and τ levels. In cell-free, cell-based and in silico studies, quercetin showed the ability to suppresses Aβ synthesis [135].

Although quercetin is able to inhibit Aβ toxicity in vitro and in vivo, the detailed mechanisms are still elusive. Other than Aβ synthesis, quercetin induces inhibition of the formation and extension of Aβ fibrils, and also stimulates and destabilizes the preformed Aβ fibrils [136,137]. The initial protein–protein interaction of Aβ_40_ and Aβ_42_, which has been proven to be necessary for Aβ oligomerization, occurs with the interference of quercetin-3-O-glucuronide [138]. Quercetin exerts neuroprotective effects against toxic molecules [139], modulating the mechanisms of cell death, increasing the resistance of neurons to oxidative stress and excitotoxicity [140], inhibiting iNOS, regulating the expression of COX-2, and exerting anti-inflammatory activity [141].

Studies have reported that several signaling pathways that participate in AD pathogenesis, such as cAMP-response element binding protein (CREB), c-Jun N-terminal kinases, the mitogen-activated protein, macroautophagy, calcium homeostasis, proteasomal degradation, and GADD34-eIF2α-ATF4 pathways, may be modulated by quercetin and its metabolites [139,142]. Quercetin also plays a role as a sirtuin-1 (SIRT-1) agonist and AChE inhibitor to ameliorate AD phenotypes [143]. Quercetin, in acting as an antioxidant, may produce a protective effect in AD and oxidative stress-related neurodegenerative diseases.

The literature shows that epigallocatechin-3-gallate (EGCG) may reduce the risk of various neurodegenerative diseases. EGCG produces neuroprotective activity by modulating mitogen-activated protein kinase (MAPK), Akt, protein kinase C, and α-secretases [144], and affecting amyloid precursor protein (APP) processing through action on the non-amyloidogenic α-secretase and the β-secretase pathways [145].

Aβ-induced cytotoxicity could be overcome by either the activation of the Akt signaling pathway [146] or by increasing the levels of acetylcholine in the presence of EGCG, which behaves as an acetylcholinesterase inhibitor. Neuroprotection upon Aβ-induced neuronal apoptosis could be achieved by effective removal of ROS. The rescue of the neuronal cells from τ-induced neurotoxicity is possible as EGCC has the capacity to remold existing oligomers to an unfolded monomeric state [147,148].

Dietary intake of EGCG, due to biological activities and mostly to antioxidant properties, has been extensively studied for its potential beneficial effects in AD. Oligonol, composed of catechin-type monomers and proanthocyanidin oligomers, is a polyphenolic compound derived from grape seed or lychee fruit with antioxidant and anti-inflammatory activities. Studies have suggested that the antioxidant effects of oligonol are directly or indirectly associated with the activation of SIRT1. Oligonol may downregulate mRNA expression related to iNOS, COX-2, NF-κBp65, and oxidative stress. Since oxidative stress and inflammatory processes are mainly associated with many neurodegenerative diseases, oligonol may have a protective effect for neurodegeneration through its activity upon oxidative-stress-induced inflammation [148].

## 5. Conclusions

AD is a common disease among aging individuals, being the sixth leading cause of all death and one of the most common causes of impairment, and about 60–80% of cases of dementia are caused by this disease. One possible method of delaying and/or preventing the onset of AD is by acting on its modifiable risk factors, amongst which diet plays an important role.

Since the rate of wine consumption is constantly increasing, numerous studies have been conducted to evaluate if it might represent a modifiable risk factor for cognitive impairment, but the results have been conflicting. Excessive wine consumption, associated with adverse brain outcomes, increases the risk of dementia by direct neurotoxic effects; however, light to moderate wine consumption seems to reduce the risk of dementia and cognitive decline in an age-dependent manner. An emerging body of literature contends that wine consumption may serve as a protective factor for cognitive decline and has associated the health properties of wine with polyphenolic content and their antioxidant properties. The increase in wine consumption is associated with factors that, in turn, promote the onset of dementia, such as hypertension and diabetes. Thus, the protection, attenuation, or intensification of AD may be based on the amount and frequency of wine consumption, individual characteristics, and individual lifestyles. Thus, further research is needed to clarify and comprehensively understand the effect of wine consumption on AD.
